# Vinorelbine-loaded multifunctional magnetic nanoparticles as anticancer drug delivery systems: synthesis, characterization, and in vitro release study

**DOI:** 10.3762/bjnano.15.24

**Published:** 2024-02-28

**Authors:** Zeynep Özcan, Afife Binnaz Hazar Yoruç

**Affiliations:** 1 Yildiz Technical University, Faculty of Chemistry and Metallurgy, Department of Metallurgical and Materials Engineering, 34210, Istanbul, Turkey

**Keywords:** drug efficacy, iron oxide nanoparticles, photothermal, solvothermal method

## Abstract

In this study, a multifunctional therapeutic agent combining chemotherapy and photothermal therapy on a single platform has been developed in the form of vinorelbine-loaded polydopamine-coated iron oxide nanoparticles. Vinorelbine (VNB) is loaded on the surface of iron oxide nanoparticles produced by a solvothermal technique after coating with polydopamine (PDA) with varying weight ratios as a result of dopamine polymerisation and covalent bonding of thiol-polyethylene glycol (SH-PEG). The VNB/PDA/Fe_3_O_4_ nanoparticles have a saturation magnetisation value of 60.40 emu/g in vibrating sample magnetometry, which proves their magnetisation. Vinorelbine, which is used as an effective cancer therapy agent, is included in the nanocomposite structure, and in vitro drug release studies under different pH conditions (pH 5.5 and 7.4) and photothermal activity at 808 nm NIR laser irradiation are investigated. The comprehensive integration of precise multifunctional nanoparticles design, magnetic response, and controlled drug release with photothermal effect brings a different perspective to advanced cancer treatment research.

## Introduction

Cancer is a widespread condition characterized by the uncontrolled proliferation of aberrant cells, which can spread to diverse body regions, encompassing over a hundred distinct forms [1–2]. Current cancer treatments lack a complete approach, as they mostly rely on radiotherapy, chemotherapy, immunotherapy, and surgery in clinical environments [3]. While these methodologies provide therapeutic benefits, they also contribute to cancer progression by inducing cytotoxicity in healthy cells and weakening the immune system, rendering individuals more vulnerable to other ailments [4–5]. There is a must to develop alternative multifunctional methodologies or intelligent drug delivery systems to formulate more effective cancer treatments, thereby addressing the current limitations encountered within this field of study. Functional nanostructures have been designed to mitigate potential harm to healthy tissue caused by these techniques [6]. Additionally, they facilitate passive targeting and offer multimodal tumor therapy.

In recent years, the use of nanotechnology-based cancer drugs has emerged as a promising alternative treatment approach. Utilizing various nanostructures as specific vehicles for drug delivery enhances efficacy and pharmacokinetic properties of anticancer drugs while mitigating the adverse effects of large dosage administration [6–7]. Additionally, it offers several advantages, such as controlled release, targeted drug delivery, and improved stability [8]. Moreover, nanoscale drug delivery systems hold great promise for specific cancer treatments, as they increase permeability and retention effect in solid tumors, enabling precise application to the targeted cells. Various structures such as silica-based conjugates, inorganic polymers, ceramic nanomaterials, gold, iron oxide, and noble metal nanoparticles have been utilized [9–10]. Among the nanostructures employed, particular emphasis has been placed on iron oxide (Fe_3_O_4_) nanoparticles. The biocompatibility and low toxicity of Fe_3_O_4_ nanoparticles have garnered significant attention in magnetic drug delivery for cancer diagnosis and treatment, primarily because of their magnetic properties [11–12]. The crystal structure of Fe_3_O_4_ nanoparticles can be tailored to allow for precise control, and these nanostructures find utility in various production processes. Magnetite nanoparticles exhibit superparamagnetic behavior due to the negligible energy barrier in the hysteresis of the particles’ magnetization cycle, as Bloch and Neel theorized [11,13]. Superparamagnetic iron oxide nanoparticles for drug delivery, diagnosis, and cancer therapy have gained wider acceptance in biomedical applications [14]. They have received notable attention in clinical applications such as early disease diagnosis (e.g., cancer, diabetes, and atherosclerosis), magnetic resonance imaging (MRI), targeted drug delivery, photothermal therapy, gene therapy, and molecular and cellular monitoring [15–16]. Photothermal therapy (PTT), a treatment in which nanostructures are used, induces drug release or damages tumor cells with the heat produced by nanostructures under NIR laser irradiation [17–18]. Compared to traditional treatments, photothermal therapy allows for increased drug release and is less cytotoxic to healthy tissues [19]. It is a minimally invasive technique that offers the advantage of rapid recovery [20]. Many well-designed agents have been developed for photothermal therapy, including carbon, metal, and organic nanocomposites [21]. Due to their superparamagnetic and heating potential, Fe_3_O_4_ nanoparticles have recently garnered attention, particularly in photothermal therapy research. Dopamine (DA) is a neurotransmitter naturally occurring in the brain and can spontaneously polymerize into polydopamine (PDA) under alkaline conditions without oxidants [22]. Polydopamine can form a coating with biocompatibility advantages, achieving up to 40% photothermal conversion efficiency, nanoscale dimensions, and customizable morphology [23–24]. Additionally, the photothermal conversion efficiency of PDA, PDA concentration, reaction time, and PDA thickness can be adjusted. Importantly, PDA exhibits a 40% photothermal conversion rate with excellent photothermal stability, indicating its significant potential as a NIR laser-driven photothermal agent [25]. However, it is challenging to completely eradicate solid tumors using PTT alone because of light scattering and limited absorption in tumor tissues. For this purpose, various modifications have been employed for passive tumor targeting. PEGylation, which involves the use of poly(ethylene glycol) (PEG) polymer, is a widely used modification method to improve passive tumor targeting and retention [26–28]. In a study presented in the literature, PEGylation was used to impart passive tumor targeting properties to PDA nanoparticles. In in vivo experiments where the synthesized nanostructure was exposed to NIR light, SN38-loaded nanoparticles effectively suppressed tumor growth chemotherapeutically and photothermally [29]. This promising result highlights the potential of the PEGylation of PDA nanoparticles for advanced cancer treatment strategies. Vinorelbine (VNB), a chemotherapeutic agent, has seen significant clinical use in the treatment of lung cancer and advanced breast cancer [30]. VNB affects the continuous mitotic division in cancer cells, thereby impeding uncontrolled growth. By binding to microtubules, VNB exerts an inhibitory effect on cancer cell growth, slowing their proliferation and disrupting mitotic regulation, leading to the stimulation of the tumor suppressor gene p53 and the inhibition or inactivation of various signaling pathways [31–32]. Its widespread adoption in medicine can be attributed to its strong therapeutic efficacy. The application of vinorelbine tartrate is limited because of its dose-related toxicity to the nervous, pulmonary, and gastrointestinal systems and reduced absorption when taken orally [33]. Encapsulation studies specifically aim to create a controlled drug delivery system to reduce existing side effects of cancer drugs or to significantly increase clinical compliance.

Zhao et al. synthesized vinorelbine-loaded and RGD-functionalized polydopamine-coated Fe_3_O_4_ superparticles via thermal decomposition [34]. Our study utilizes a solvothermal method to synthesize nanostructures with a spherical morphology and a size of 18 nm. After coating with PDA at different ratios, the size reaches up to 28, 61, and 225 nm. Another point is that PEGylation has been applied using SH-PEG polymer to enhance biocompatibility. Notably, our study demonstrates a significantly higher drug loading efficiency of 98%, indicating the superior efficacy of our synthesis method. Moreover, our drug-loaded nanostructures exhibit a saturization magnetization of *M*_s_ = 60.40 emu/g, highlighting enhanced magnetic properties compared to the cited study. This indicates that the nanostructure can be strongly manipulated under an external magnetic field. This finding is crucial for future studies on magnetic field-guided drug release and tumour treatment. Particularly, our research also investigates the effect of varying polymer ratios on drug release kinetics and photothermal efficiency, which was not addressed in the abovementioned paper. It was demonstrated through this study that as the amount of PDA polymer increased, both photothermal heating efficiency and drug release decreased, while the drug release rate increased when photothermal heating was applied. Fe_3_O_4_ nanoparticles with adjustable magnetic properties and appropriate sizes exhibited controlled drug release capabilities. Thus, a controlled drug delivery system was established using VNB/PDA/Fe_3_O_4_ NPs, which exhibited high release at the tumor microenvironment pH 5.5 for potential application in cancer treatment. The impact of polymer thickness on drug release was also determined.

Consequently, our study represents a novel contribution to the field by investigating the impact of polymer thickness on drug release, offering enhanced drug loading efficiency, improved magnetic properties, and pH-responsive drug release kinetics.

## Materials and Methods

### Materials

The chemicals used in nanoparticle synthesis, namely iron(III) chloride hexahydrate (FeCl_3_·6H_2_O, *M*_w_ = 270.30 g/mol), iron(II) chloride tetrahydrate (FeCl_2_·4H_2_O, *M*_w_ = 198.81 g/mol), dopamine hydrochloride (*M*_w_ = 189.64 g/mol), tris(hydroxymethyl)aminomethane hydrochloride, (Tris-HCl, *M*_w_ = 157.60 g/mol), and thiol-polyethylene glycol (SH-PEG, purity: ≥95%), ammonium hydroxide (NH_4_OH, 28–30%), phosphate-buffered saline (PBS) were purchased from Merck. The chemical vinorelbine tartrate CRS (European Pharmacopoeia (EP) Reference Standard, catalog no: Y0000463) was also acquired from Merck.

### Synthesis of Fe_3_O_4_ nanoparticles

The starting materials, 3 g of FeCl_3_·6H_2_O and 1.5 g of FeCl_2_·4H_2_O, were dissolved in ethylene glycol under magnetic stirring for 30 min. Ammonium hydroxide solution (28–30%) was gradually added (approximately 5 mL, until the color of the solution changed from orange to black) [35–36]. Subsequently, the prepared mixture was placed in an autoclave and subjected to thermal treatment at 200 °C for 6 h. After the heat treatment process, Fe_3_O_4_ NPs were separated from the liquid using a magnet, and the produced Fe_3_O_4_ NPs were dried in a vacuum oven at 60 °C for 24 h.

### Coating of Fe_3_O_4_ nanoparticles with polydopamine

The synthesized Fe_3_O_4_ NPs were weighed (100 mg) and dispersed in 50 mL of Tris-HCl solution (10 mM, pH 8.5) [37]. The dispersion process was carried out using a magnetic stirrer. Dopamine hydrochloride was then added to the produced solutions at various ratios (dopamine hydrochloride amounts: 100, 200, and 400 mg; dopamine hydrochloride/Fe_3_O_4_ ratios: 1:1, 2:1, and 4:1) and stirred at room temperature for 15 h at 1000 rpm. Following this stage, the obtained PDA-coated Fe_3_O_4_ NPs were separated from the solution using a magnet. Subsequently, unreacted material was removed by washing three times with distilled water, and the PDA/Fe_3_O_4_ NPs were dried in a vacuum oven at 55 °C.

### Surface functionalization of PDA/Fe_3_O_4_ nanoparticles with SH-PEG

For the surface modification process with SH-PEG, 50 mg of PDA/Fe_3_O_4_ NPs were added to 50 mL of Tris-HCl solution. Then, 100 mg of thiol-polyethylene glycol was added to the prepared mixture to homogenize the solution. Subsequently, 0.2 mL of ammonium hydroxide (NH_4_OH, 28–30%,) was added, and the mixture was stirred at room temperature for 70 min [37]. SH-PEG-modified PDA/Fe_3_O_4_ NPs were separated from the solution using a magnet, and unreacted particles were removed by washing with distilled water. The PEGylation PDA/Fe_3_O_4_ NPs were then dried in a vacuum oven at 45 °C.

The PDA-coated Fe_3_O_4_ nanoparticles were modified with SH-PEG to facilitate their accumulation in tumour regions. In similar studies, the conjugation of SH-PEG onto the surface of PDA polymer was achieved through the Michael addition reaction, involving the thiol and carbonyl groups present in the SH-PEG structure [37–38].

### Vinorelbine loading on PDA/Fe_3_O_4_ nanoparticles

For loading vinorelbine tartrate into the nanostructure, 25 mg of PEGylation PDA/Fe_3_O_4_ nanoparticles (1 mg/mL) were combined with 25 mL of phosphate solution (pH 8.5). The purpose of this mixture was to facilitate the loading of vinorelbine tartrate into the PDA/Fe_3_O_4_ content. Subsequently, 25 mg of VNB was added to the prepared mixture, and the solution was thoroughly mixed for 5 h. The resulting nanostructure was separated with the assistance of a magnet, followed by three thorough rinses with distilled water. All wash supernatants were collected to measure the VNB loading content based on UV–vis spectrophotometry. The resulting nanostructure underwent vacuum drying at 45 °C. The loading entrapment efficiency (%EE) [39] of VNB into Fe_3_O_4_ NPs was calculated using Equation 1; it was found that the entrapment efficiency was approximately 98%.


[1] $$EE\left(\%\right)=\frac{\text{initial drug amount}-\text{unentrapped drug}}{\text{total drug amount}}\times 100$$

### Standard curve of vinorelbine

The calibration curve for the time-dependent release of VNB was generated by preparing VNB solutions at concentrations of 0, 20, 40, 60, 80, 100, 200, and 400 µg/mL. These solutions were then placed in quartz cuvettes, and absorbance readings were taken using the UV–vis spectrophotometer at a wavelength of 268 nm [40]. The absorbance values obtained were utilized to construct the calibration curve.

### Determination of photothermal stability and efficiency

Fe_3_O_4_ NPs, PDA/Fe_3_O_4_ NPs, and VNB/PDA/Fe_3_O_4_ NPs (at a concentration of 0.1 mg/mL and in a total volume of 1 mL) were exposed to 808 nm (1 W/cm^2^) NIR laser irradiation for a duration of 5 min. PBS was used as a control. The temperature changes of the NP solutions were recorded using an infrared thermal imaging camera. Additionally, the photothermal stability of both PDA/Fe_3_O_4_ NPs and VNB/PDA/Fe_3_O_4_ NPs (at a concentration of 0.1 mg/mL and in a total volume of 1 mL) was assessed through a 5 min interaction with an 808 nm (1 W/cm^2^) NIR laser followed by a cooling process, for four cycles [41].

### Determination of vinorelbine drug release

VNB/PDA/Fe_3_O_4_ NPs (at ratios of 1:1, 2:1, and 4:1) were placed into dialysis capsules at a concentration of 1 mg/mL. Subsequently, each prepared dialysis capsule was placed in 100 mL phosphate solution at pH 5.5 and 7.4 [30]. The experiment was conducted at 37 °C with a shaking speed of 150 rpm. The experiment involved obtaining a 1 mL sample at specified time intervals (0.5, 1, 2, 3, 4, 5, 6, 12, 24, 30, 36, 48, and 50 h). The sample was then analyzed using a UV–vis spectrophotometer. The mean values of the results obtained in triplicate were taken. The concentration of the drug release was calculated using Equation 2 [42] with the calibration curve for VNB.


[2] $$\begin{array}{c} \text{concentration of drug }\left(\mu \text{g}/\text{mL}\right)\\ =\left(\text{slope}\times \text{absorbance}\right)\pm \text{intercept} \end{array}$$

In the in vitro dissolution test, the drug release (DR) was determined using Equation 3.


[3] $$\begin{array}{c} \text{DR }\left(\text{mg}/\text{mL}\right)\\ =\left(\begin{array}{l} \text{concentration}\times \text{dissolution bath volume}\\ \times \text{ dilution factor} \end{array}\right)/1000 \end{array}$$

The cumulative percentage of drug release (CPR %) was determined using Equation 4 [42] separately for pH 5.5 and 7.4. Furthermore, the cumulative drug release percentages of VNB/PDA/Fe_3_O_4_ NPs prepared with different ratios were compared to investigate the effect of the PDA ratio on VNB drug release.


[4] $$\begin{array}{c} \text{CPR }\left(\%\right)=\\ \frac{\text{the volume of samples withdrawn}\left(\text{mL}\right)\times P_{\left(t-1\right)}+P_{t}}{\text{bath volume}\left(v\right)}, \end{array}$$

where *P_t_* is the percentage release at time *t* and *P*_(_*_t_*_–1)_ is the previous percentage release.

### Determination of photothermal-responsive drug release

The dialysis capsules were exposed to an 808 nm (1 W/cm^2^) NIR laser for 5 min at specific time points (15, 30, 45, 60, 120, 240, and 300 min) to assess the impact of laser irradiation on drug release. The drug content in VNB/PDA/Fe_3_O_4_ NPs (at ratios of 1:1, 2:1, and 4:1) and the cumulative drug release were determined by calculating according to Equations 2–4 utilizing the absorbance values obtained from UV–vis spectrophotometer and calibration curves.

### Characterization

The morphology of the synthesized nanoparticles was determined with a high-resolution analytical electron microscope (FE-SEM, Thermo Scientific, Apreo 2S LoVac) and a scanning transmission electron microscope (STEM, Phillips XL, 30 ESEM-FEG/EDAX) operating at 120 kV acceleration voltage. The structure of the nanoparticles was analyzed by X-ray diffraction (XRD, PANalytical, Xpert Pro) using Cu Kα radiation (λ = 0.15418 nm) in a 2θ range of 10° to 90°. Fourier-transform infrared (FTIR, Thermo, Nicolet Is 10) spectra of the nanostructures were obtained in the range of 4000–400 cm^−1^.

The amounts of released drug were obtained using a UV–vis spectrophotometer (Shimadzu Scientific Instruments, UV-1800) at a wavelength of 268 nm. Magnetic properties of nanoparticles were evaluated by vibration sample magnetometry (VSM, Lake Shore, Model 7410) using field-induced magnetization measurements at 298 K. The average diameters of nanoparticles were determined using ImageJ (US National Institute of Health, Bethesda, MD, USA). OriginPro 8.5 (OriginLab, MA, USA) was used for statistical analysis. A *p*-value less than 0.05 (*p* < 0.05, *n* = 4) was considered statistically significant.

## Results and Discussion

### Characterization of Fe_3_O_4_ nanoparticles

**Figure 1:**
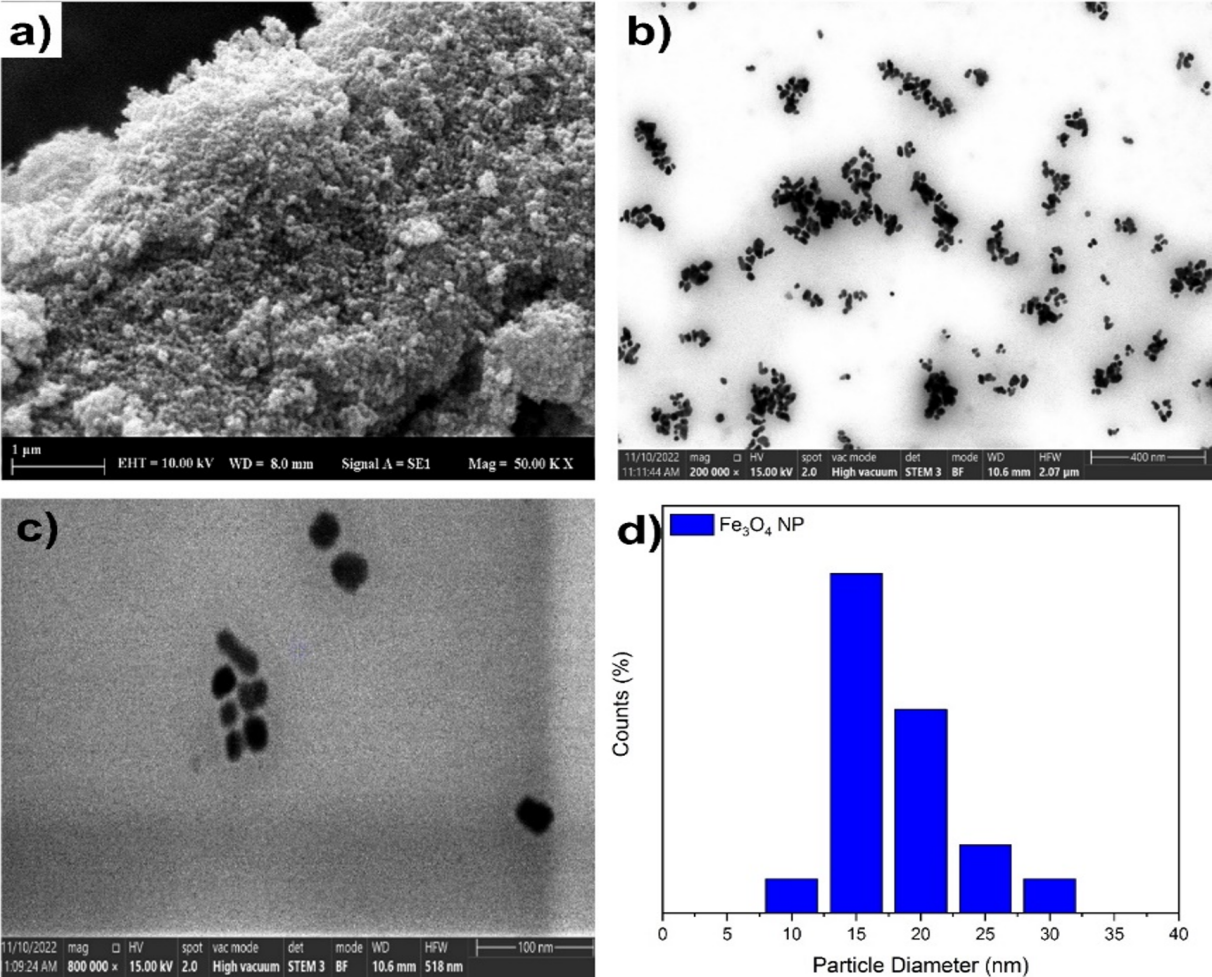
(a) FE-SEM images of Fe_3_O_4_ NPs. (b) STEM images of Fe_3_O_4_ NPs at 200,000× magnification. (c) STEM images of Fe_3_O_4_ NPs at 800,000× magnification. (d) Particle diameter distribution of Fe_3_O_4_ NPs.

The synthesis of Fe_3_O_4_ NPs was carried out using a solvothermal technique in a stainless steel reactor at 200 °C for 6 h. According to the results of FE-SEM and STEM examinations, the Fe_3_O_4_ NPs are spherical, as depicted in Figure 1a–c. When examining the STEM size distribution, it was observed that Fe_3_O_4_ NPs were efficiently synthesized with an average size of 18 nm.

The Fe_3_O_4_ NPs consist of 99.9% magnetite and have a cubic reverse spinel structure. Magnetite exhibits a spinel crystal structure resulting in a face-centered cubic arrangement in which oxygen atoms are positioned opposite the other constituent atoms. The Fe_3_O_4_ NP (311) reflection shows a significantly wide full width at half maximum, indicating the presence of ultrafine particles and a small crystal size. The crystal size was determined using the Scherrer equation (Equation 5) [43] applied to the most prominent diffraction peaks of Fe_3_O_4_ NPs.


[5] D=K∗λ/(β∗cosθ)

Equation 5 shows the relationship between peak broadening and particle size in X-ray analysis. In this equation, the symbols *D*, *K*, λ, β, and θ represent the particle size, Scherrer shape factor (here 0.89), X-ray wavelength (0.15418 nm), half-maximum width, and diffraction angle, respectively [43]. Using the X-ray diffraction (XRD) spectrum and Equation 5, the particle size of Fe_3_O_4_ NPs was calculated and determined to be 18 nm on average.

**Figure 2:**
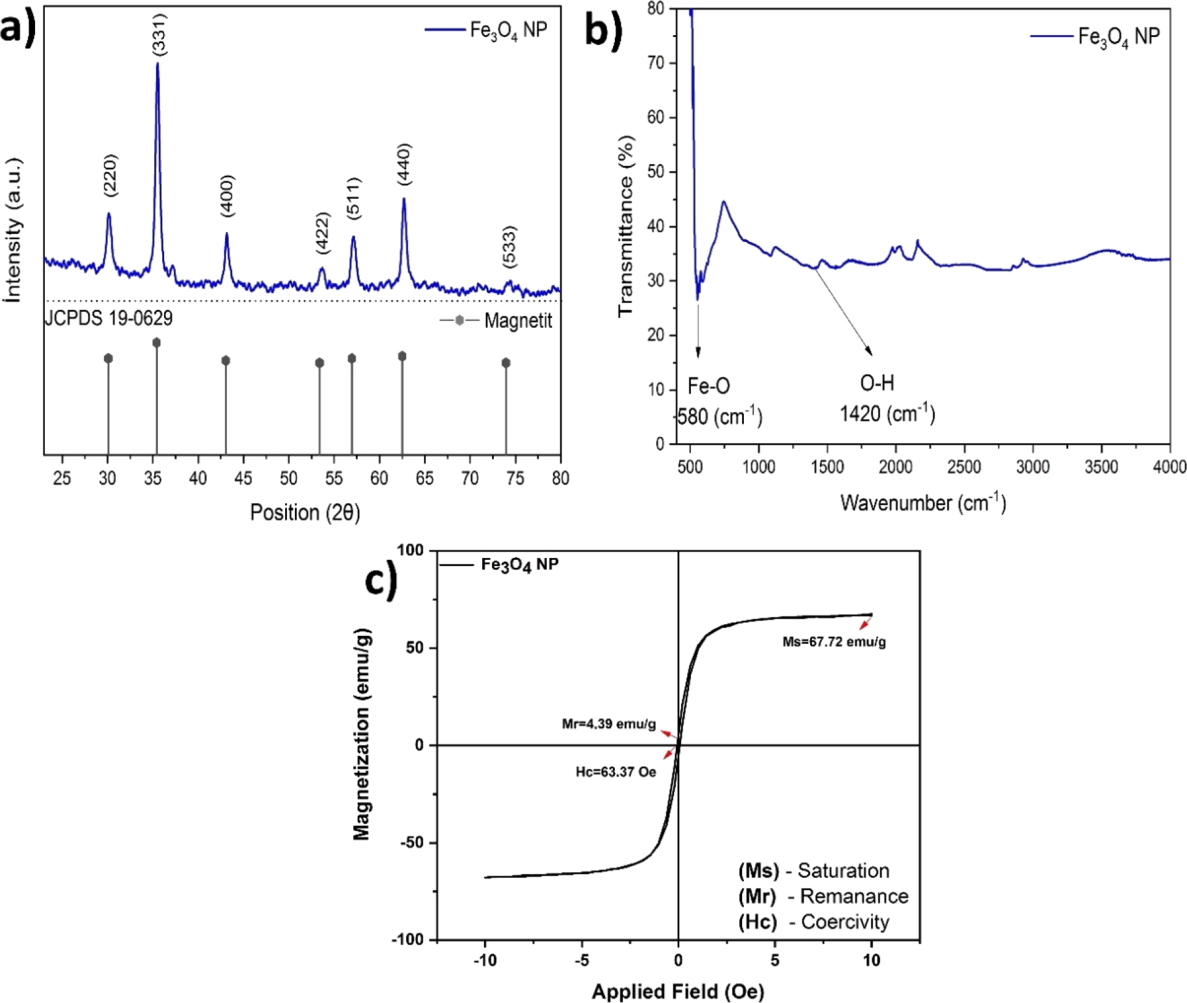
(a) X-ray diffraction patterns of Fe_3_O_4_ NPs. (b) FTIR spectra of Fe_3_O_4_ NPs. (c) Hysteresis loop for Fe_3_O_4_ NPs.

X-ray patterns showing the distribution of Fe_3_O_4_ NPs in their uncoated state are shown in Figure 2a. XRD analysis reveals the presence of seven distinct peaks at 30.13°, 35.48°, 43.12°, 53.6°, 56.08°, 62.7°, and 73.92°. These peaks can be assigned to the corresponding crystallographic planes of magnetite Fe_3_O_4_: (220), (311), (400), (422), (511), (440), and (533), respectively. Fe_3_O_4_ NPs exhibit a peak consistent with the data obtained for the reference ICDD no. 19-629 [43].

The FTIR spectra presented in Figure 2b show the characteristic peak associated with the Fe–O bond of Fe_3_O_4_ NPs at a wavenumber of 580 cm^−1^. The peak detected at a wavenumber of 1420 cm^−1^ was attributed to –OH groups in Fe_3_O_4_ NPs [44]. A vibrating sample magnetometer (VSM) was used to investigate the magnetic properties of the Fe_3_O_4_ NPs [45]. Various factors such as the crystal structure of the material, dimensions, morphology, and density of crystal defects significantly affect the magnetic properties [46]. The saturation magnetization (*M*_s_) values of NPs measured at 298 K using a VSM are given in Figure 2c. The values obtained for saturation magnetization (*M*_s_), coercivity (*H*_c_), and residual magnetization (*M*_r_) were determined as 67.72 emu/g, 63.37 Oe, and 4.39 emu/g, respectively.

### Characterization of polydopamine coating, PEGylation, and drug loading modifications of Fe_3_O_4_ nanoparticles

The Fe_3_O_4_ NPs were synthesized using a solvothermal method. Subsequently, Fe_3_O_4_ NPs were coated with PDA in different ratios. The coating process involved the use of PDA in ratios of 1:1, 2:1, and 4:1, respectively. PDA can undergo polymerization resulting in the adsorption onto the surface of the negatively charged Fe_3_O_4_ NPs [22]. During this process in an alkaline environment, PDA polymerizes into its oxide form. As a result, the nanostructure undergoes coating with PDA [47]. The average distribution of PDA coating sizes and thicknesses was determined using FE-SEM size analysis.

**Figure 3:**
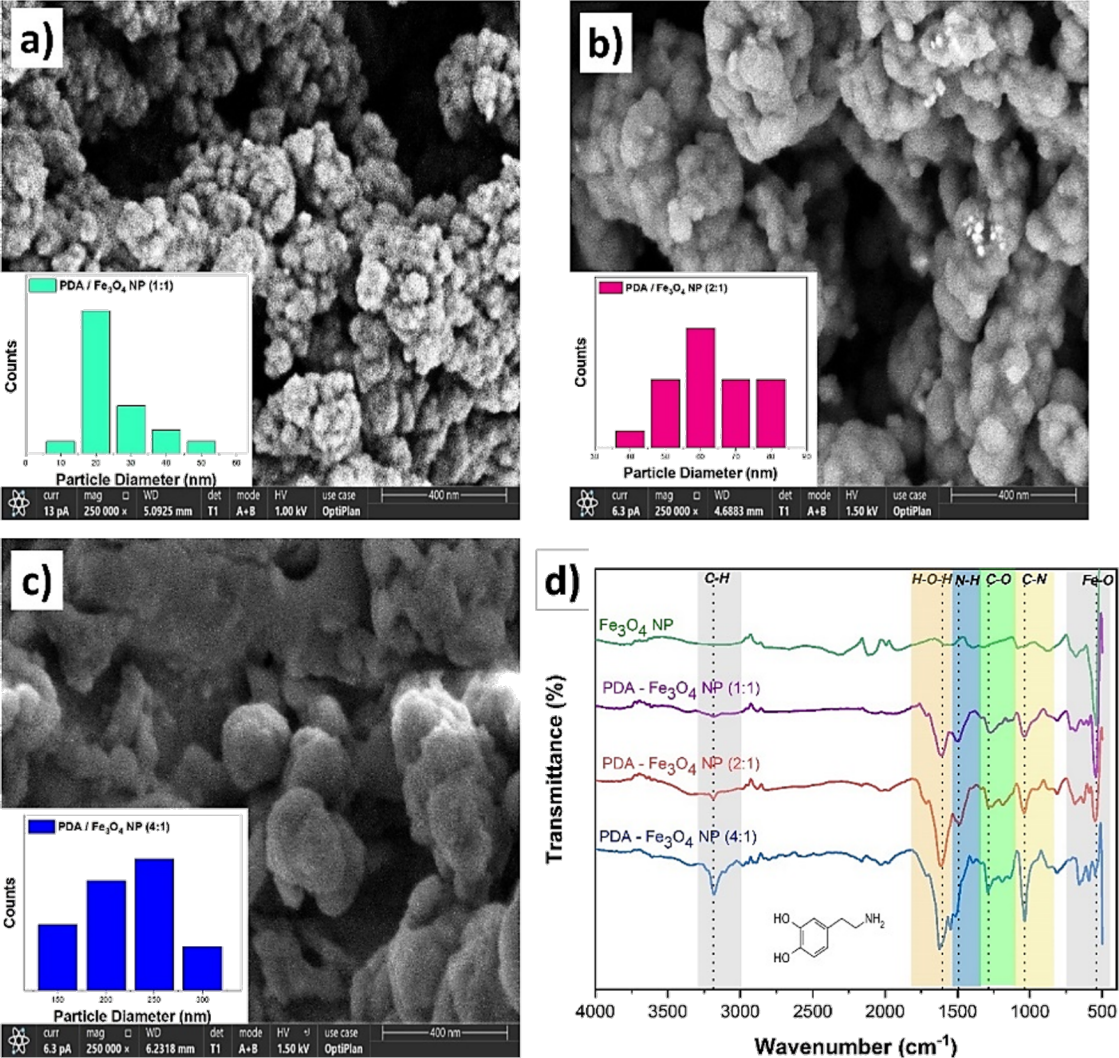
FESEM images of (a) PDA/Fe_3_O_4_ NPs (1:1), (b) PDA/Fe_3_O_4_ NPs (2:1), and (c) PDA/Fe_3_O_4_ NPs (4:1), all 250,000× magnification. (d) FTIR spectra of Fe_3_O_4_ NPs and PDA/Fe_3_O_4_ NPs (1:1, 2:1, and 4:1 ratio).

In Figure 1c, the average size of bare Fe_3_O_4_ NPs was 18 nm, whereas in Figure 3a, after PDA coating (1:1 ratio), the average size of Fe_3_O_4_ NPs was 28 nm. Hence, it is postulated that Fe_3_O_4_ NPs have been subjected to 10 nm PDA coating. Figure 3b shows the PDA/Fe_3_O_4_ NPs with 2:1 ratio demonstrating an average size of 61 nm and an average coating thickness of 43 nm. In a similar vein, it can be observed from Figure 3c that the PDA/Fe_3_O_4_ NPs (4:1 ratio) exhibit an average size of 225 nm, corresponding to a coating thickness of 103.5 nm. The FTIR spectra of Fe_3_O_4_ NPs and PDA/Fe_3_O_4_ NPs (1:1, 2:1, and 4:1) are given in Figure 3d. The absorption peaks at 587 and 1620 cm^−1^ are indicative of Fe_3_O_4_ NPs. These results are in line with a study by Xue and co-workers [45]. The broad absorption bands in the 1700–1000 cm^−1^ range suggest the presence of aromatic rings and phenolic compounds in PDA. These bands demonstrate the effective coating with PDA, as illustrated in Figure 3d.

A study by Feng et al. noted a peak at 1259 cm^–1^ in the IR spectra of PDA/Fe_3_O_4_ NPs, attributed to the extension vibration of the C–O band. The obtained results are supported by the resemblance to the peak observed at 1221 cm^−1^ in this observation. Furthermore, the peaks observed at 1520 and 1595 cm^–1^ can be attributed to the stretching vibration of C–O units, which is further supported by the peak at 1221 cm^–1^ [48].

Studies in the literature have demonstrated that the coating of iron oxide nanoparticles, commonly employed in creating multifunctional particles with the capability of passive targeting in magnetic fields for photothermal cancer therapy, with PDA holds great promise for future applications. Therefore, surface modification with PDA is recognized as a favorable alternative for enhancing the biocompatibility of non-biodegradable substances.

**Figure 4:**
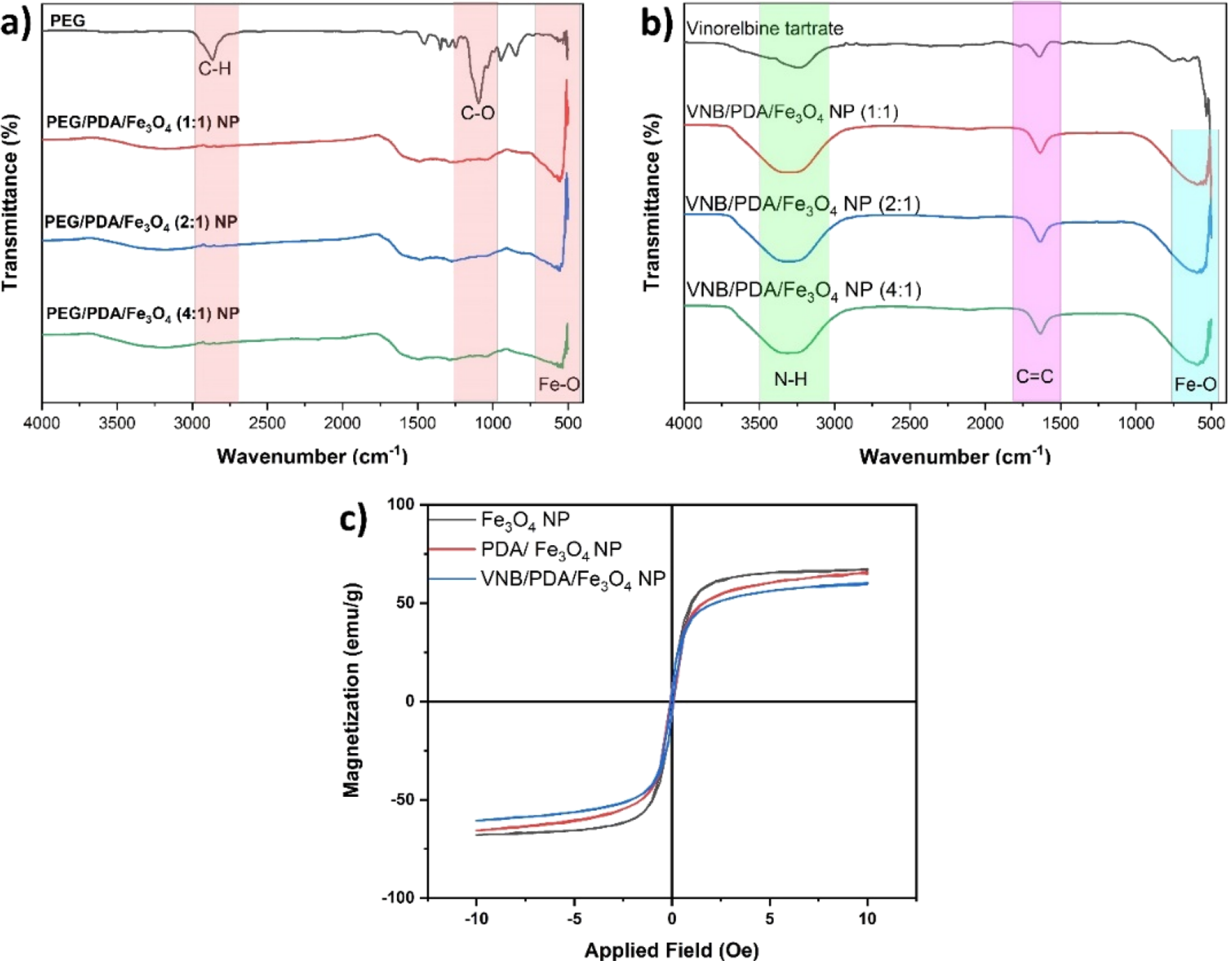
(a) FTIR spectra for Fe_3_O_4_ NPs and PDA/Fe_3_O_4_ NPs. (b) FTIR spectra for VNB and VNB/ PDA/Fe_3_O_4_ NPs. (c) Hysteresis loops for Fe_3_O_4_ NPs, PDA/Fe_3_O_4_, and VNB/PDA/Fe_3_O_4_ NPs.

A study focused on examining the binding of PEG to PDA/Fe_3_O_4_ NPs and the resulting chemical structure using FTIR spectroscopy. According to the FTIR analysis results, the peak at 585 cm^–1^ in the spectrum corresponds to the vibration associated with the Fe–O bond in magnetic nanoparticles [49]. The peak observed at 3400 cm^–1^ can be attributed to the vibration associated with stretching hydroxy (–OH) groups in Fe_3_O_4_ NPs (Figure 4a). FTIR analysis revealed that the peaks observed at 1150 and 2890 cm^–1^ correspond to the vibrations associated with stretching C–O–C and C–H groups in SH-PEG, respectively [50]. The presence of band structures at 1500 and 1000 cm^–1^ in the FTIR analysis of SH-PEG provides evidence for the surface modification of PDA/Fe_3_O_4_ NPs with SH-PEG. The FTIR analysis results indicate the successful functionalization of PDA/Fe_3_O_4_ NPs with SH-PEG molecules. This will enable the passively targeted delivery of the created nanoplatform to tumor tissues and enhance biocompatibility. Similar studies have described conjugated PEG–iron oxide nanoparticles as multifunctional nanotherapeutic agents for passive targeting of tumors [49–50]. Additionally, in the literature, it has been demonstrated that multifunctional PEGylated magnetic nanoparticles coated with polydopamine (PDA) exhibit strong near-infrared absorption because of the PDA layer and have the ability to deliver drugs under a magnetic field owing to their superparamagnetism [51].

During the drug loading studies, the anticancer drug vinorelbine was incorporated in the structure of PDA/Fe_3_O_4_ NPs during the polymerization of dopamine. It was observed that a significant portion of VNB in VNB/PDA/Fe_3_O_4_ NPs was absorbed within the polymer shell, while a small portion was retained on the surface [52]. According to FTIR analyses, drug-related features are visible in the nanostructures as N–H peaks located at 3500–3000 cm^−1^ (Figure 4b). This result demonstrates the effective incorporation of VNB into the nanostructure. As a result, the VNB compound exhibits a prominent peak attributed to the presence of a carbon–carbon (C–C) group at 1573 cm^−1^ and a nitrogen–hydrogen (N–H) group in the range of 3500–3000 cm^−1^ [53]. The FTIR spectrum of VNB/PDA/PDA/Fe_3_O_4_ NPs displays all PDA, SH-PEG, and VNB peaks, indicating the successful formation of a core–shell structure containing these three components.

According to the VSM analysis, the saturation magnetization of Fe_3_O_4_ NPs was 67.72 emu/g; PDA/Fe_3_O_4_ NPs had a saturation magnetization of 65.62 emu/g; VNB/PDA/Fe_3_O_4_ NPs showed a saturation magnetization of 60.40 emu/g, as shown in Figure 4c. The observed decrease in magnetization is commonly attributed to the polymer coating on the surface of the magnetic nanoparticles [49]. Based on the findings from VSM, the nanoparticles exhibit high magnetization [49,54]. The magnetic properties of VNB/PDA/Fe_3_O_4_ NPs can be attributed to the structural arrangement of Fe_3_O_4_ within the nanoparticles. A magnetic field can enhance the dispersion of VNB/PDA/Fe_3_O_4_ NPs in an aqueous solution, showing promising prospects for use in magnetically targeted therapy.

### Determination of photothermal stability and efficiency

**Figure 5:**
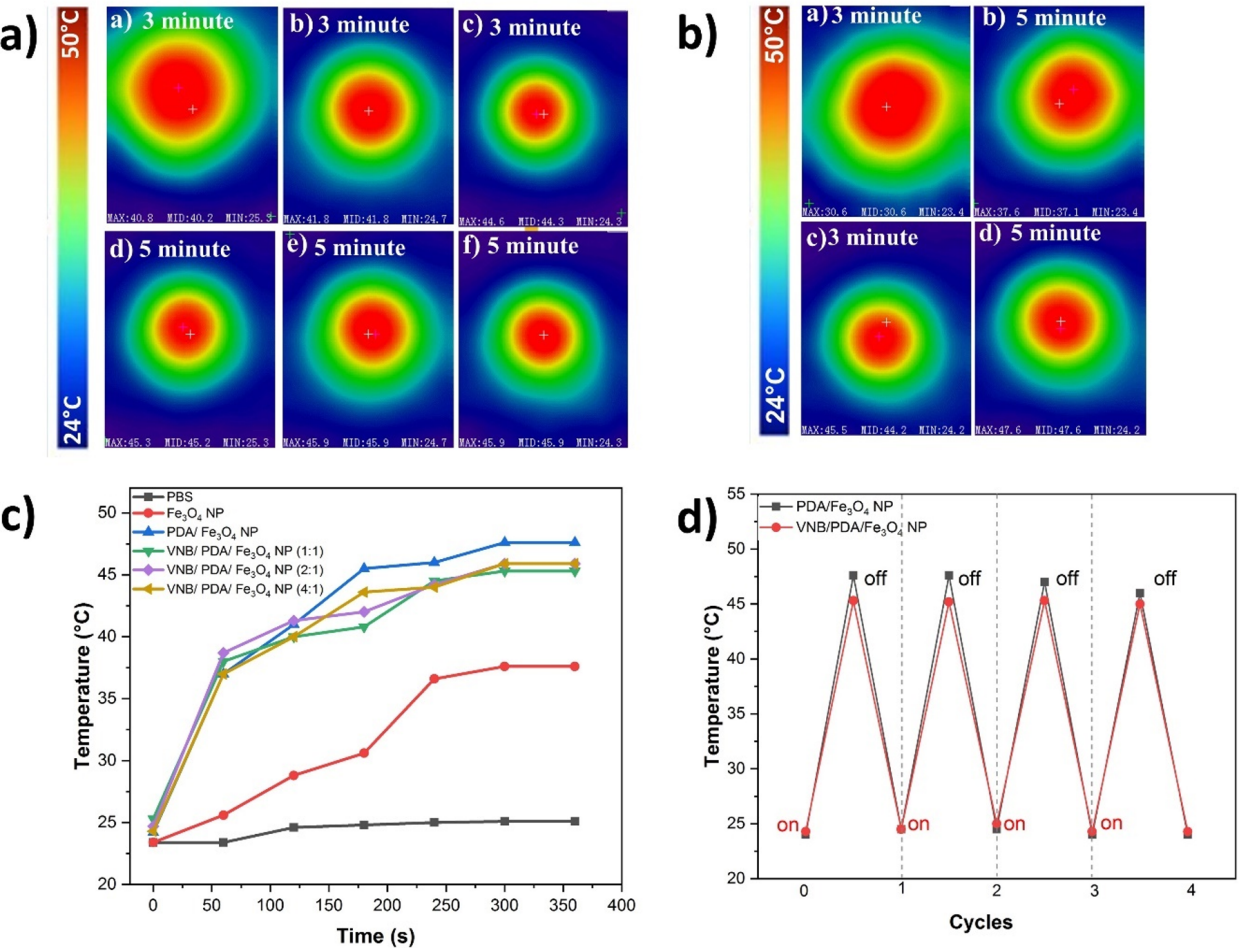
(a) Infrared thermal camera images of NPs at ratios of 1:1, 2:1, and 4:1, respectively, after 808 nm laser (1 W/cm^2^) irradiation: (a, d) VNB/PDA/Fe_3_O_4_ NPs at a 1:1 ratio, (b, e) VNB/PDA/Fe_3_O_4_ NPs at a 2:1 ratio, and (c, f) VNB/PDA/Fe_3_O_4_ NPs at a 4:1 ratio. (b) Infrared thermal camera images of (a, c) Fe_3_O_4_ NPs and (b, d) PDA/ Fe_3_O_4_ NPs after 808 nm laser (1 W/cm^2^) irradiation. (c) Temperature changes during laser interaction. (d) Cyclically repeated temperature changes.

To evaluate the photothermal performance of the nanostructures, the NPs were irradiated with an 808 nm laser at a power density of 1 W/cm^2^ for 5 min. A slight increase in temperature was observed in the phosphate-buffered saline (PBS, control) solution. As shown in Figure 5b, when exposed to NIR laser, the temperature of the Fe_3_O_4_ NP solution reached a maximum of 37.6 °C.

In contrast, Figure 5b(b,d) illustrates a rapid increase in the temperature of PDA/Fe_3_O_4_ NPs, reaching a peak at 47.6 °C after 5 min. This swift temperature rise is likely attributed to the enhanced stability of PDA in PDA/Fe_3_O_4_ NPs and its higher NIR absorption capabilities. The temperature of VNB/PDA/Fe_3_O_4_ NPs (at ratios of 1:1, 2:1, and 4:1) varied between 45.3 °C and 45.9 °C after 5 min of laser irradiation. All drug-loaded nanostructures reached a heating temperature of 40 °C after 3 min and did not exceed a maximum temperature of 46 °C. These findings indicate a promising potential for applying these nanostructures in photothermal therapy. Figure 5c presents the results of the photothermal efficiency study for PDA/Fe_3_O_4_ and VNB/PDA/Fe_3_O_4_ NPs. Following four cycles of NIR laser irradiation, the photothermal stability of the NPs was maintained, with only a negligible decrease observed. These results demonstrate that local hyperthermia induced by NIR laser irradiation can be precisely controlled in an on/off mode, showcasing sensitivity to temperature-induced drug release [41].

### Determination of cumulative and photothermal-responsive drug release

The calibration curve was constructed by plotting the UV–vis absorbance values against the amounts of VNB solution generated at various concentrations. The generated calibration curve was utilized to determine the cumulative drug release and encapsulation efficiency. Equation 6 represents the calibration curve derived from absorbance readings:


[6] $$y=0.01716x-0.0888\;\left(R^{2}=0.9942\right)$$

The entrapment efficiency of VNB onto the polydopamine-coated iron oxide nanoparticles (PDA/Fe_3_O_4_ NPs) is approximately 98%. The loading of VNB is limited to the interior and surface of the PDA shell, excluding the Fe_3_O_4_ core. Therefore, a significant amount of VNB is associated with the drug loading rate and the entrapment efficiency within the PDA shell.

For the determination of cumulative drug release, VNB/PDA/Fe_3_O_4_ NPs were examined over a period of 50 h in acidic citrate buffer at pH 5.5 to simulate the tumor microenvironment. The NPs were also studied in pH 7.4 PBS to ascertain the drug release profile. The cumulative drug release rates (%) for VNB/PDA/Fe_3_O_4_ NPs (1:1, 2:1, and 4:1) are presented in Table 1.

**Table 1:** Cumulative drug release.

Cumulative drug release (%) for VNB/PDA/Fe_3_O_4_ NPs						

Time (h)	pH 5.5			pH 7.4		
	
	1:1	2:1	4:1	1:1	2:1	4:1

0.5	21.58042	22.6993	24.58741	24.64336	24.64336	13.37063
1	27.15087	22.21381	24.58706	31.31503	31.31503	34.78252
2	27.01136	23.11731	25.92552	34.45787	34.45787	36.85157
3	29.13164	26.33636	27.37483	35.76101	35.76101	37.99003
4	29.86171	27.07465	28.65647	37.92832	37.92832	39.87832
5	31.16993	27.52448	30.1458	40.84458	40.84458	40.21801
6	33.91661	29.57028	60.81031	41.66573	41.31923	44.58392
12	77.04266	59.38584	61.93759	56.25245	55.47488	50.09965
24	84.56853	68.91241	63.93889	57.71789	55.93342	52.46066
30	98.1993	78,52115	68.75017	57.15261	61.90699	53.66883
36	98.56853	79.43177	71.85297	61.41678	61.64126	54.90997
48	99.59126	81.32038	73.93889	61.85332	64.30839	54.66883
50	99.63636	81.91801	75.79266	67.2743	64.12168	55.09965

According to the data in the Table 1, VNB/PDA/Fe_3_O_4_ (1:1) NPs exhibited the maximum release in pH 5.5 citrate buffer. VNB/PDA/Fe_3_O_4_ (1:1) NPs demonstrated a release efficiency of 99% within a 50 h timeframe in pH 5.5 citrate buffer. These NPs exhibited a maximum release rate of 77% within the initial 12 h, reaching 94% after 24 h. At pH 7.4, after 50 h, a rate of 67% was observed. In contrast, NPs with VNB/PDA/Fe_3_O_4_ ratios of 2:1 and 4:1 exhibited release efficiencies of 81% and 75% at pH 5.5, respectively.

Compared to a similar study [34], our findings reveal significant differences in drug release kinetics; our nanostructures exhibit a higher drug release percentage at pH 5.5 (84.57%) compared to pH 7.4 (57.71%). This underscores the pH-responsive behaviour of our drug delivery system, which could potentially enhance drug delivery to tumour sites while minimizing off-target effects.

### Determination of photothermal drug release

**Figure 6:**
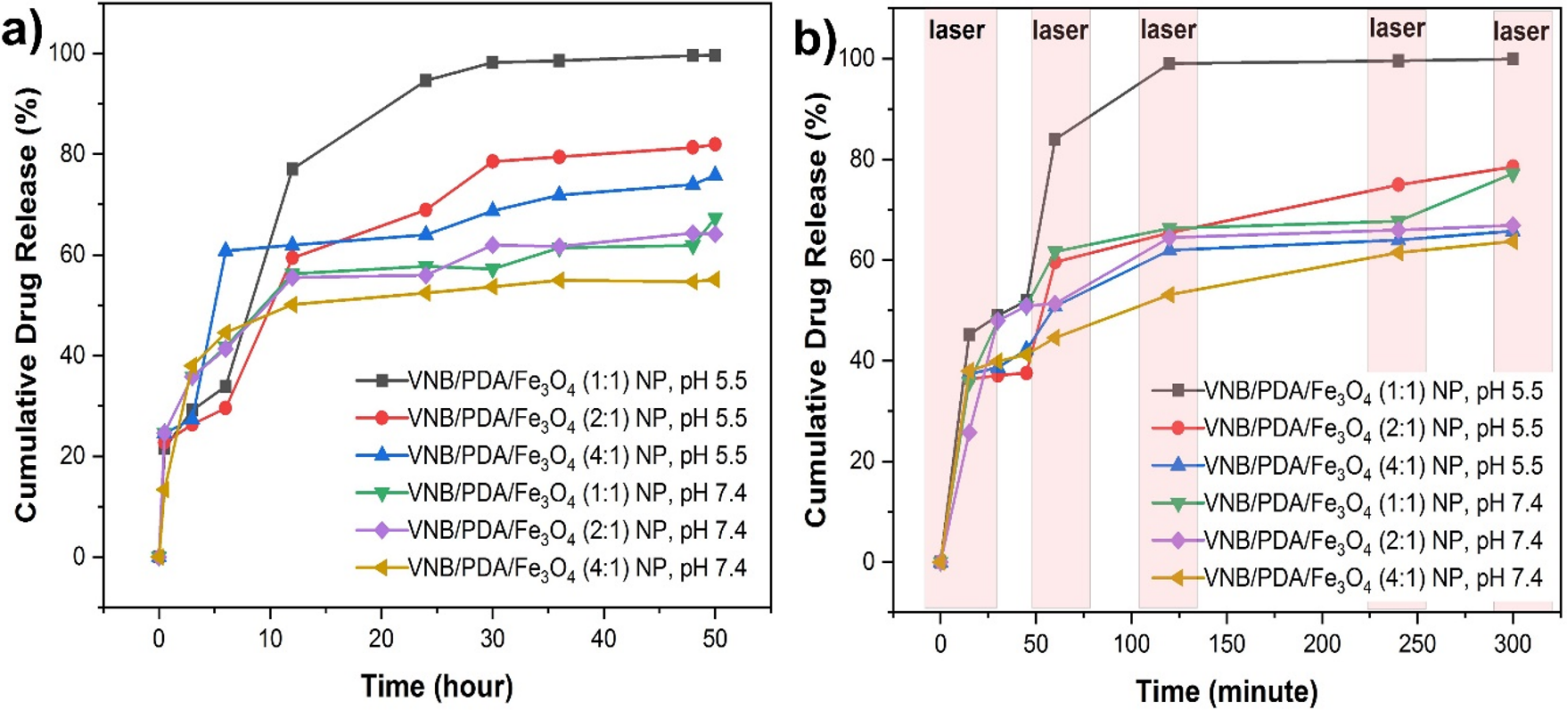
(a) Cumulative drug release of VNB/PDA/Fe_3_O_4_ NPs (1:1, 2:1, and 4:1 ratios). (b) Photothermal drug release of VNB/PDA/Fe_3_O_4_ NPs (1:1, 2:1, and 4:1 ratios).

To investigate the effect of NIR laser interaction on drug release, VNB/PDA/Fe_3_O_4_ NPs were irradiated with an 808 nm NIR laser at a power density of 1 W/cm^2^ for 5 min at different time intervals (15, 30, 45, 60, 120, 240, and 300 min). After exposing VNB/PDA/Fe_3_O_4_ NPs (1:1) to 5 min of NIR irradiation, 46.16% of VNB was released, as illustrated in Figure 6b. The drug release was significantly increased to 99.04% after 120 min. The exposure of the VNB-loaded nanostructure to NIR irradiation resulted in enhanced drug release due to the heightened temperature induced by the thermosensitive Fe_3_O_4_ NPs. Furthermore, the enhanced heating due to the photothermal properties of PDA facilitated the separation of VNB from the structure [45]. As shown in Figure 5c, it is worth noting that the temperature of PDA/Fe_3_O_4_ NPs increased from 25 to 45 °C in 3 min following the NIR irradiation and reached 47.6 °C after 5 min.

Regarding the drug release from laser-irradiatied VNB/PDA/Fe_3_O_4_ NPs (2:1), there was a 68% increase in drug release within the initial 12 h, with 81% of the drug being released within 50 min at pH 7.4; 64% release was observed after 50 h. For VNB/PDA/Fe_3_O_4_ (4:1), possessing the highest PDA ratio, the drug release was 75% at pH 5.5 and 55% at pH 7.4. The observed drug release rates of VNB/PDA/Fe_3_O_4_ NPs (specifically, at ratios 2:1 and 4:1) reaching a maximum of 78% after 300 min is intriguing (Table 2).

**Table 2:** Laser interaction and cumulative drug release for VNB/PDA/Fe_3_O_4_ NPs.

Cumulative drug release (%) VNB/PDA/Fe_3_O_4_ NPs						

Time (mn)	pH 5.5			pH 7.4		
	
	1:1	2:1	4:1	1:1	2:1	4:1

15	49.16113	36.33636	37.37483	35.76101	25.76101	37.99003
30	49.86170	37.07465	38.65647	47.92832	47.92832	39.87832
45	51.99116	37.52448	42.14580	50.84458	50.84458	41.21801
60	83.91663	59.57028	50.81031	61.66573	51.31923	44.58392
120	99.04267	65.38584	61.93759	66.25245	64.47488	53.09965
240	99.56859	74.91241	63.93889	67.71789	65.93342	61.46066
300	99.3210	78.52115	65.75017	77.15261	66.90699	63.66883

One possible explanation for this phenomenon lies in the thickness of the PDA coating. It is plausible to consider that as the PDA layer becomes thicker, it may pose a barrier to efficient heating of the Fe_3_O_4_ core. This could result in a delayed release compared to formulations with a thinner PDA layer.

Moreover, it’s worth noting that a thicker polymer layer may impede surface erosion. This aspect is crucial in drug release, as it can lead to a slower, more controlled release of the encapsulated drug [55]. Thus, the thickness of the PDA coating emerges as a pivotal factor influencing the release dynamics of the loaded drug [56]. Another critical aspect that warrants attention is the potential impact of different pH values on the oxidative capacity of the coating material polydopamine. This coating, formed through the polymerization of dopamine, plays a pivotal role in the drug delivery system. The variations in pH values can potentially modulate the chemical environment in which the polymerization occurs [57].

Consequently, pH alterations may induce changes in the surface charge of the nanoparticles. This could profoundly affect the drug binding capacity of the nanoparticles and implies that the nanoparticles may exhibit varying affinities for the drug molecule at different pH values. Hence, the observed differences in drug release profiles between pH 5.5 and 7.4 can be plausibly attributed to these pH-dependent interactions [57]. Factors such as pH value, coating material properties, coating thickness, and drug binding capacity significantly influence drug release [58].

Based on the presented findings, it can be concluded that polymer thickness and NIR laser irradiation affect the drug release process. When externally applied, NIR laser irradiation can facilitate the release of VNB from VNB/PDA/Fe_3_O_4_ NPs and induce a photothermal interaction at the tumor site. The synthesized VNB/PDA/Fe_3_O_4_ NPs hold promise for effective photothermal therapy, magnetic targeting, MRI imaging, and chemotherapeutic capabilities in future studies.

## Conclusion

In this study, we successfully synthesized VNB/PDA/Fe_3_O_4_ NPs with combined photothermal therapy and chemotherapy functionalities using a solvothermal method. The incorporation of PDA into the fabricated nanostructures imparts several advantages for cancer therapy and controlled drug release systems because of its robust structural and physicochemical properties. Additionally, aside from enhancing photothermal therapy capabilities, the PDA shell mitigates nanomaterial toxicity while increasing biocompatibility. The strategic integration of PEGylation into tumor-targeted drug delivery systems significantly amplifies passive tumor targeting and retention through the enhanced permeability and retention effect, thereby enhancing its efficacy in cancer treatment. Furthermore, our findings underscore the pivotal roles played by polymer thickness, the acidic tumor microenvironment, and NIR laser irradiation in the drug release process. Notably, the application of a NIR laser in conjunction with the acidic tumor microenvironment triggers the controlled release of VNB. When combined with laser-induced photothermal therapy, this results in effective tumor elimination without recurrence. This mechanism holds immense promise for precise and targeted drug delivery.

Moreover, VNB/PDA/Fe_3_O_4_ NPs exhibit noteworthy potential in photothermal therapy, magnetic targeting, MRI imaging, and chemotherapy. This versatile approach represents a significant advancement in cancer treatment modalities, offering a promising avenue for future research and clinical applications. Our work provides a nanomaterial endowed with dual-targeting capabilities for the synergistic treatment of cancer via photothermal and chemotherapy, demonstrating excellent application prospects in the future.

## Data Availability

The data that supports the findings of this study is available from the corresponding author upon reasonable request.
